# Latent Semantic Analysis Discriminates Children with Developmental Language Disorder (DLD) from Children with Typical Language Development

**DOI:** 10.1007/s10936-018-09625-8

**Published:** 2019-01-25

**Authors:** Rasmus Bååth, Sverker Sikström, Nelli Kalnak, Kristina Hansson, Birgitta Sahlén

**Affiliations:** 1 Lund University Cognitive Science, Lund University, Lund, Sweden; 2 Department of Psychology, Lund University, Lund, Sweden; 3 Department of Clinical Sciences, Lund, Logopedics, Phoniatrics and Audiology, Lund University, 221 85 Lund, Sweden; 4 Department of Women’s and Children’s Health, Astrid Lindgren Children’s Hospital, Karolinska Institutet, Stockholm, Sweden

**Keywords:** Developmental language disorder, Latent semantic analysis, Semantic linguistic ability, Narratives

## Abstract

Computer based analyses offer a possibility for objective methods to assess semantic-linguistic quality of narratives at the text level. The aim of the present study is to investigate whether a semantic language impairment index (SELIMI) based on latent semantic analysis (LSA) can discriminate between children with developmental language disorder (DLD) and children with typical language development. Spoken narratives from 54 children with DLD and 54 age matched controls with typical language development were summarized in a semantic representation generated using LSA. A statistical model was trained to discriminate between children with DLD and children with typical language development, given the semantic vector representing each individual child’s narrative. The results show that SELIMI could distinguish between children with DLD and children with typical language development significantly better than chance and thus has a potential to complement traditional analyses focussed on form or on the word level.

## Introduction

Traditionally, most studies and the focus of assessment methods for developmental language disorder (DLD, earlier labeled specific language impairment, Bishop et al. 2017) have been on form (phonology or grammar) and lexical or semantic skills at the word level. Furthermore, earlier studies have not focused on objective methods to quantify semantic content at text the level, possibly due to lack of appropriate methods to measure this. In a recent study, Hansson et al. (2016) introduced a method, based on Latent Semantic Analysis (LSA, Landauer and Dumais 1997), which provides opportunities to quantify semantic linguistic maturity in children’s narratives with the aim of providing insights into semantic development. This method first generates a high dimensional semantic representation of words that are created from the co-occurrence of words in a large text corpus. This representation was applied to predict the chronological age from children with typical language development (aged 4–13) in a task where the children generated spoken narratives from pictures. In the present study we propose that this LSA-based method can be extended to discriminate between children with DLD and children with typical language development. This may be important because children with DLD have difficulties that are limited to language development and typical development in other areas, i.e. without other conditions that differentiates them from children with typical development (Bishop et al. 2017). DLD varies in severity and profile of language difficulties and is characterized by deficits in aspects of receptive and expressive language, as well as communication. The prevalence of DLD in preschool children has been estimated to 6–7% (Law et al. 2000; Norbury et al. 2016; Tomblin et al. 1997).

### Semantic Skills in Children with Developmental Language Disorder

An early sign of DLD is late and slow vocabulary development (Alt et al. 2004; McGregor et al. 2013; Nash and Donaldson 2005) and need for more exposures for word learning (Rice et al. 1994). In school age, a substantial proportion of children with DLD are described to have difficulties with both lexical production and reception, e.g. word-finding problems (Dockrell et al. 1998; German and Simon 1991; Leonard et al. 1983) and weak semantic representations (Kail and Leonard 1986; McGregor and Appel 2002; McGregor et al. 2002). In addition, semantic deficits have also been shown in studies of comprehension at the phrase, sentence and text levels (Bishop 1997; Bishop and Adams 1992; Norbury 2004).

Sheng and MacGregor (2010) assessed naming skills in children with and without DLD. The children with DLD were slower and less accurate in naming tasks than age matched controls, although naming skills were as expected given their vocabulary level. Naming errors suggest immature semantic representation in DLD according to the authors. Thus, the main problem in children with DLD may not be a small vocabulary size, but immature semantic representations or missing/weaker links between representations within the semantic network. The idea is that the stronger the links between representations in the network, the easier the words are accessed. The networks are created during development when children “learn about words through their co-occurrence with other words” (Corrigan 2008: 109). The meaning of a word emerges from all information from the contexts where a child has heard or read this word, thus creating semantic networks.

It is of great importance not to focus only on word level skills, but also analyse language skills at the text level, using narratives. Narrative tasks (story retelling, picture elicited narratives or personal narratives) are important in clinical assessment of children with DLD, since they provide information about a range of skills related to linguistic form, content and use. As argued by Botting (2002), a large body of research has been carried out on narrative development in children with typical language development and has shown that narrative development starts early and developmental patterns have been documented, i.e., normative data are available. There is also evidence that narrative skills are representative of language use in other contexts and narrative skills are also associated with literacy. Furthermore, this is a task where children with language disorder have documented difficulties. Their narratives are more similar to those of younger children, than to those from same age peers (Botting 2002). Furthermore, narratives represent a more naturalistic task than language tests, but still more controlled than data obtained during spontaneous conversations. Fey et al. (2004) point to the importance of analysing narratives on both the macro and micro level. The macro level refers to story content and organization (here as rating of overall quality). The micro level refers to the use of grammatical and lexical features. Typical measures are number of utterances, mean length of utterance and number of different words as well as analysis of language details like grammatical accuracy and use of complex structures. In their study Fey et al. (2004) found that macro structure and grammatical accuracy best distinguished between children with DLD and controls, whereas the length measures (mean length of utterance and number of utterances) did not consistently distinguish the groups. In a study of narratives from children with DLD and children with autism spectrum disorders Norbury and Bishop (2003) also analysed both on the macro and micro level. Their findings also corroborate the usefulness of narratives for assessment of language skills and an important conclusion was that core language abilities are more important for narrative skills than pragmatic skills or diagnostic status.

Studies on lexical-semantic skills at the text level can be based on spoken and written narratives or conversational speech samples and is most often measured as lexical diversity. Several different measures have been used to capture lexical diversity and to evaluate their diagnostic accuracy in comparisons between children with DLD as well as other clinical groups and multilingual children and controls. Some measures are more straightforward to compute, like number of different words (Bedore et al. 2010; Hansson et al. 2000; Kover et al. 2012; Simon-Cereijido and Gutiérrez-Clellen 2009; Watkins et al. 1995), number of different verbs (Reuterskiöld et al. 2010; Simon-Cereijido and Gutiérrez-Clellen 2009) and type-token ratio (McCarthy and Jarvis 2010). Other measures are computer based and more complex, since they are based on mathematical models that control for the effect of sample length. Examples are the Giraud index (McCarthy and Jarvis 2010), the measure D (Asker-Árnason et al. 2012; Klee et al. 2004; McCarthy and Jarvis 2010; McKee et al. 2000; Ooi and Wong 2012; Owen and Leonard 2002) and Measure of Textual Lexical Diversity (MLTD; McCarthy and Jarvis 2010).

Implications for efficient use of narrative sample analysis in clinical protocols for semantic skills need more investigation. The novelty of the present study is that we focus on a measure of the quality of semantic content in narratives, not specifically semantic diversity, and the potential clinical application of such a measure.

### Semantic Spaces

To develop a model that can quantify semantic content in children’s narratives we have applied a method from the field of computational linguistics called *semantic spaces*. A semantic space analysis places the words of a text corpus into a multidimensional space in such a way that words that are semantically similar are found closer together than semantically dissimilar words. For example, the word *puppy* is expected to be found close to the word *dog* but far from the similar sounding, but semantically distant, *puppet*. Using a constructed semantic space, it is possible to quantify the semantic similarity between different words and, by extension, the semantic similarity between different texts. One of the most prominent methods for creating semantic spaces is Latent Semantic Analysis (LSA; Landauer and Dumais 1997). Furthermore, LSA has been proposed as a general theory of semantic knowledge representation to explain and simulate how semantic representations of words are acquired (Landauer and Dumais 1997). LSA is a “high-dimensional associative model” of semantics (Landauer and Dumais 1997: 211), presupposing no other knowledge than a general learning mechanism. It is based on induction and assumes that we create the meaning of words through hearing/reading them in contexts together with other words. This creates an explanation of how we can know so much general information of the world based on the scarce information we get (Landauer and Dumais 1997). Models, like LSA, have been found to be useful for extracting meaning at the text level (McNamara 2011). The LSA algorithm is highly data-driven and does not use syntactic information such as word order or what word class a word belongs to, and when applied to a text corpus it produces a high dimensional semantic space where each word is represented as a vector in this space. What number of dimensions to use can be determined by choosing the number of dimensions that provides the highest quality of the space. This quality can be measured by a synonym test, that is, by looking at the rank order of the semantic closeness between two synonymous words, and where such semantic representation exist for the Swedish language (e.g., Karlsson et al. 2013). Typically, optimal performance is found when the number of dimensions are in the order of hundreds (Dumais 1992).

In a recent study (Hansson et al. 2016), LSA was used on narratives from Swedish-speaking children to generate a measure of *semantic linguistic maturity* (SELMA) based on a semantic representation of words. The semantic representation was created from the co-occurrence of words in a large text corpus on spoken narratives from children with typical language development in the age range 4–11. By comparing the SELMA measure with maturity ratings made by speech-language pathology students, the length of the narratives, and the age of the children, SELMA was found to have higher agreement with the maturity ratings made by the speech-language pathology students than with chronological age and number of words. One conclusion was that it is possible to use quantitative measures for studying the development of lexical- semantic development in children’s narratives. Already this indicates that the method could also be used to identify children with DLD, given that their performance is often similar to younger peers (e.g., Botting 2002). However, children with DLD do not perform exactly like younger peers in narratives. One important aspect to take into account is that with increased age children have more linguistic experiences and general knowledge than younger peers, which might affect their lexical-semantic skills. There is a need for the development of tools that can differentiate children with DLD from children with typical language development with respect to lexical-semantic skills, with a focus on not only vocabulary breadth, but also vocabulary depth.

### Aim

The aim of the present study is to investigate how well a semantic language impairment index (SELIMI) based on LSA can discriminate between Swedish-speaking children with DLD and children with typical development on the basis of their spoken narratives.

## Method

### Participants

The participants were 54 children with DLD aged 98–153 months. All children had a non-verbal IQ above 70, only few performed below IQ 80. The children with DLD represent two different samples from studies of DLD in Swedish populations. One sample, group A, consisted of 36 children with DLD of non-specified severity attending main stream schools (Asker-Árnason et al. 2012; Hansson et al. 2004). The criteria for these participants when recruited for research projects at pre-school age were performance at least one standard deviation below the mean for their age on the grammatical subtest of a Swedish test of expressive language abilities (Holmberg and Stenkvist 1983) in spite of normal hearing, non-verbal IQ within the normal range and otherwise typical development. The other sample, group B, consisted of 18 children with severe DLD attending school language units (Kalnak et al. 2012). Criteria for admission to such schools are normal hearing, non-verbal above IQ 70, no autism spectrum disorder or intellectual disability and that they perform substantially below norms on tests of language production and language comprehension. The children met all these criteria, as assessed by a team consisting of a speech-language pathologist, a psychologist and a special teacher at age 6–7, when they were admitted to the special school. A new assessment was made at age 8–12 for inclusion in the project and confirmed their DLD status.

Thus all these participants were considered to fulfil the criteria for DLD (Bishop et al. 2017) when recruited, and all were currently receiving or had received language intervention. Within each study the DLD status was verified through clinical assessments. Due to the difference in characteristics and an age-difference (the children in group A are on average older than the children in group B) we treat them separately in the analyses (see Table 1).

**Table 1:** Group, age and gender distribution in the two sets of data

Group	N	Girls (N)	Age range (months)	Mean age (SD)
Group A				
DLD	36	18	107–153	124 (10.0)
Controls	36	17	109–153	123 (9.8)
Group B				
DLD	18	4	98–109	102 (3.7)
Controls	18	6	99–107	103 (2.6)

In addition, a control group consisting of 54 children with typical language development participated as individually age-matched controls, 36 for group A, 18 for group B. Age matching was made ± 2 months. The controls were typically developing children according to parental and teacher reports. They were recruited through mainstream schools.

All children were monolingual speakers of Swedish.

All parents had given written consent for their children to participate. The projects were approved by the Regional Ethical Review Boards in Lund and Stockholm, Sweden.

### Data Collection Procedure

The narratives were elicited using a selection of pictures from the story “One frog too many” (Mayer and Mayer 1975). The pictures were selected to represent six story grammar units suggested by Stein and Glenn (1979). The units were Setting, Initiating event, Response state, Response plan, Attempt, Consequence and Resolution/Reaction. The pictures from “One frog too many” were laid out, one at a time, and the child was asked to look carefully at each picture. Thereafter the test administrator asked the child to tell the story. The test administrator was instructed to avoid providing support apart from nodding and acknowledging by a “mhm” or a “yes”, or asking “Is that it?” in order to make sure the child was done. The procedure was audio- and video recorded and later transcribed. The transcriptions formed the basis for the analyses described below.

### Analyses

The analysis of the semantic representation used methods that have been described in detail elsewhere (e.g., Kjell et al. 2018; Landauer and Dumais 1997). Thus, here we just provide a brief overview of the method. A semantic space was created from a corpus consisting of approximately 100,000 articles taken from the 100 largest Swedish newspapers in 2007. The choice of this corpus was made due to being the largest Swedish dataset available to the research group at the time when this research was carried out. The space was created using the Infomap (http://infomap-nlp.sourceforge.net/index.html) software package. Unless otherwise specified, the default parameter settings of this software was used. First a co-occurrence matrix was generated, where the rows consisted of the 100,000 most common words, and the columns of the context of the 10,000 most commons words. The context was defined as the 15 words preceding and following the target word. This co-occurrence matrix was normalized by applying the logarithm plus one, which moderates the impact of high frequency words. A data compression algorithm called singular value decomposition (SVD) was applied to this matrix, with the aim of maintaining as much information as possible from the original matrix while reducing the number of dimensions. The resulting semantic space consisted of the 100,000 most common words in the corpus (i.e., high frequency words such as pronouns were included in the space). Each word in the space is represented by a 100-dimensional semantic vector, with a length normalized to one.

The additional semantic analyses were conducted using the SemanticExcel software (www.semanticexcel.com), an internet based research tool for statistical analysis and quantification of text data (for details on this software see Kjell et al. 2018, 2019). Furthermore, the statistical computing environment R (R-core 2016) was used. The narratives generated by the participants were summarized in the semantic representation generated by LSA. This was done by summing the semantic vectors representing the words in each narrative that also were present in the semantic space (words missing from the space were ignored), so that each narrative was summarized into a single vector consisting of the same number of dimensions as for single words. Most words in a single narrative did not occur in other narratives. However, the semantic representation generated from the large corpus allowed these words to be mapped to the same representations. The length of this vector was normalized to a length of one (i.e., the same length as the representation of each word).

### Semantic Linguistic Impairment Index (SELIMI)

We defined and fitted a statistical model that discriminates between children with DLD and children with typical language development. The input to the model was a semantic representation representing the words that children produced in the “One frog too many” stories. After being trained on the semantic representation and the status of the children the model was evaluated by testing how well it predicted the status of a child, given that child’s semantic representation. The technical details of how to set up prediction models to predict a numerical value from a semantic representation has been described elsewhere (see for example Kjell et al. 2018), so here we only provide an overview of the method. The predictive model was fitted separately for group A and for group B. The number of narratives (72 in group A and 36 in group B) used in the training was much lower than the number of predictor variables (i.e., the 100 dimensions of the semantic vectors). Therefore, we used *logistic ridge regression* (Le Cessie and van Houwelingen 1992) as the statistical model, which is a regression model that can handle binary outcome variables where the number of training examples is fewer than the number of predictor variables. The binary outcome variable was whether a narrative was produced by a child with DLD or not, and the predictors were the semantic dimensions of each narrative. To fit this model, we used the implementation available in the glmnet (Friedman et al. 2010) package available for the R statistical environment. This package uses penalized maximum likelihood as the criteria to fit the model. The result of applying a trained regression model to a novel narrative would be a score between 0.0 and 1.0 representing the estimated probability that the narrative was produced by a child with DLD. We call this score the *Semantic Linguistic Impairment Index (SELIMI).* In order to assess model fit and to evaluate the predictive performance of the model we employed a leave-one-out cross validation scheme: The SELIMI of each narrative was predicted using a regression model trained on the remaining narratives from the same group (group A and group B respectively), and by this the predictive performance when applying the model to new data was estimated. In the present study we decided for a cut-off value of .5 for the model to discriminate between children with DLD and controls. The SELIMI score can be interpreted as a probability to turn this score into a binary prediction (DLD or typical language development) we used a cut-off of .5. For example, if the SELIMI score given to a narrative was .73, then this can be interpreted as the model assigning a 73% probability to that the narrative was produced by a child with DLD and, as most of the probability is on that the narrative was produced by a child with DLD (i.e., it is higher than .5), this is the final binary prediction.

### Metrics used to Evaluate SELIMI

To evaluate the success of SELIMI to correctly identify children with and without DLD, sensitivity, specificity, positive likelihood ratios and negative likelihood ratios were calculated. Sensitivity is the proportion of children correctly identified as having DLD, i.e., the number of true positives divided by the sum of true positives and false negatives. Specificity is the number of true negatives divided by the sum of true negatives and false positives. True positives are children with a positive outcome on SELIMI who have DLD, false negatives are children with a negative outcome on SELIMI in spite of having DLD. True negatives are children with a negative outcome on SELIMI who have typical language development and false positives are children with a positive outcome on SELIMI in spite of having typical language development. A positive likelihood ratio of 10 or higher and a negative likelihood ratio of .1 or lower are required for large confidence in a measure’s ability to identify the presence (positive likelihood ratio) or absence (negative likelihood ratio) of the disorder.

## Results

**Fig. 1:**
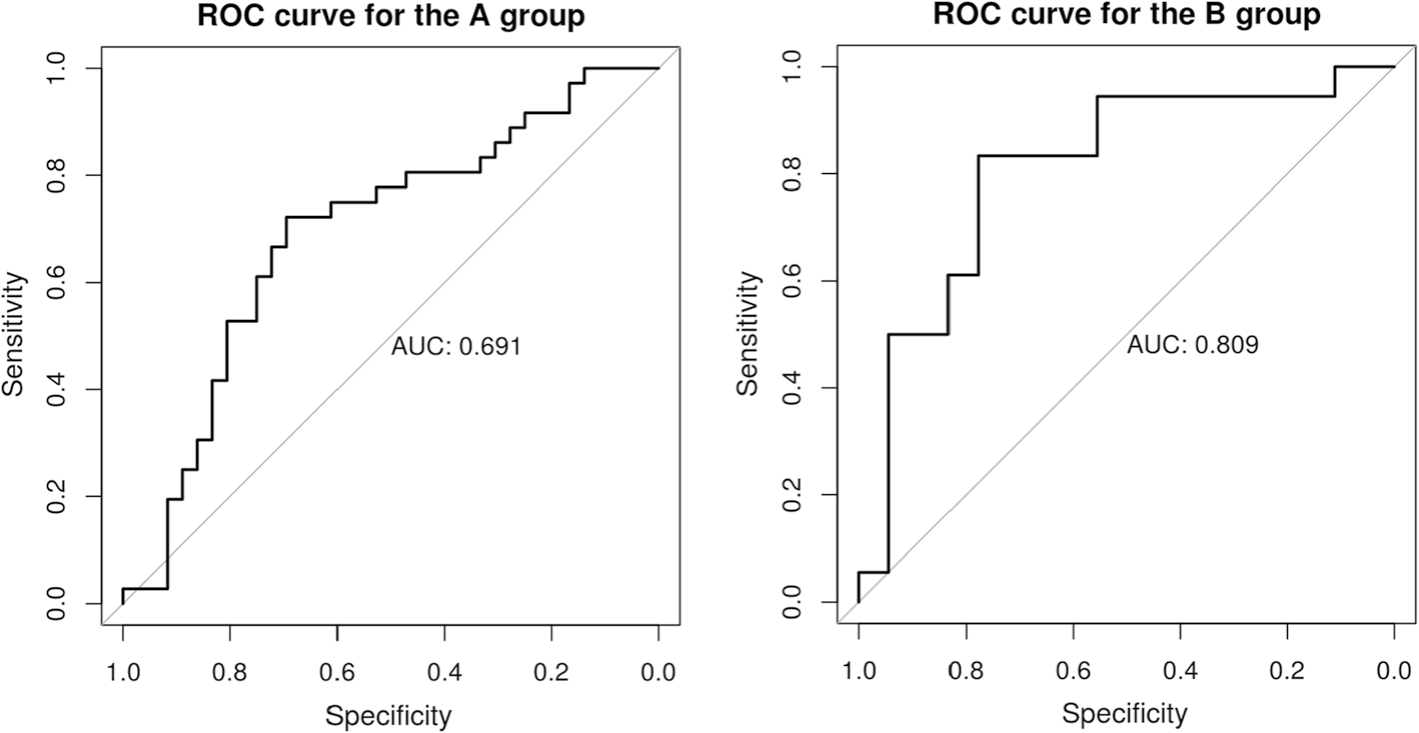
Receiver operating characteristic (ROC) curves for the A and B group. The gray line shows the chance level performance

Table 2 shows the number of true and false positives and negatives using a SELIMI cut off of .5. The sensitivity (the rate of true positives) and the specificity (the rate of true negatives) was comparable in both groups. In group A sensitivity was 64% and specificity 72% whereas in group B sensitivity was 78% and specificity was 83%. This means that the proportion of children with DLD correctly identified by SELIMI as having DLD (sensitivity) was somewhat lower than the proportion of children with typical language development correctly identified by SELIMI as such (specificity). This corresponds to a positive likelihood ratio of 2.3 and a negative likelihood ratio of 0.50 for group A, and a positive likelihood ratio of 4.6 and a negative likelihood ratio of 0.26 for group B. The positive likelihood ratios indicate slight to moderate confidence in SELIMI’s ability to identify the presence of the disorder. The negative likelihood ratios also indicate slight to moderate confidence in its ability to identify the absence of the disorder. In total, 68% of the participants were correctly predicted for group A and 81% were correctly predicted for group B as having either DLD or typical language development based on our model. A binomial test gave that these rates of success (68 and 81% respectively) were statistically significant from a null model predicting presence of DLD at random, with *p* = 0.003 for group A group and *p* < 0.001 for group B. Figure 1 shows *receiver operating characteristic* (ROC) curves for group A and group B, that is, these curves show the predictive performance for all cut-offs between 0.0 and 1.0. The area under the ROC was .691 for group A and .809 for group B.

**Table 2:** The performance of the predictive model under leave-one-out cross validation

	Group A			Group B	
	Predicted group			Predicted group	
Actual group	DLD	Control	Actual group	DLD	Control
DLD	23	13	DLD	14	4
Control	10	26	Control	3	15

## Discussion

The results indicate that the new measure SELIMI distinguished between children with a diagnosis of DLD and children with a typical language development significantly better than chance in both data sets and with a higher degree of accuracy in the B-group than in the A-group.

In group A 68% of the participants were correctly identified as either true positives or true negatives, as compared to 81% in group B. Sensitivity was 64% in group A and 78% in group B and specificity 72% in group A and 83% in group B. Thus, when using SELIMI to predict DLD the results for group A are below, or slightly below, and results for group B are close to, what has been described as acceptable levels of sensitivity and specificity (Plante and Vance 1994). However, a single tool which can correctly identify 64–78% of individuals with, and 72–83% without a complex condition like DLD merits consideration. SELIMI focuses mainly on one aspect of language skills, semantics. The interpretation of the importance of a sensitivity level must take the complexity of the clinical condition and the context for the assessment (when, how and where it was carried out) into account (see discussion below).

Dividing the participants with DLD in the present study in groups A and B was motivated by the fact that the participants in the groups differed regarding age and when DLD-status was latest confirmed. The children in group A were slightly older (mean age 10;4) and represented a large variation regarding severity of language disorder, ranging from milder to more severe problems, and they attended mainstream elementary schools. Their DLD status was not re-confirmed when participating in the present study but was based on clinical assessments and diagnosis in preschool-age. This means that some of the children in Group A could have outgrown their language disorder (Stothard et al. 1998). Children in Group B were somewhat younger (mean age 8;6), They qualified for attending school language units in preschool age because of having a severe degree of DLD and demonstrated persistent language difficulties at age 8 when participating in the present study. In spite of these differences, the difference in the levels of sensitivity and specificity were not statistically significant between group A and B. This indicates that our tool seems to work well not only in a population with more severe DLD but also in a group representing a broader span of severity. We believe that our main result, regarding SELIMI’s ability to discriminate between DLD and controls, is strengthened by the fact that the findings did not differ significantly between two different populations with DLD.

### Application of SELIMI in a Clinical Context: Challenges and Possibilities

In a clinical context children are usually assessed with more than one tool to get a broader picture of their strengths and difficulties, and formal as well as dynamic assessment procedures are advocated. Thus, a tool like SELIMI cannot constitute the only basis for diagnosis, but is expected to be used in combination with other assessment tools. Furthermore, the linguistic profile of difficulties in children with DLD varies (Reilly et al. 2014; Leonard 2014). It is not reasonable to expect higher sensitivity of SELIMI than what we found because not all children with DLD have marked difficulties in the semantic domain, and semantic limitations may not be obvious in all linguistic contexts.

It is also important to take into account the considerable changes in lexical semantic aspects in narratives during school years. Given that it is a developmental disorder, DLD has dynamic features (Hansson et al. 2014). The constellation of linguistic strengths and weaknesses within a child is not static but changes with both time and context. Language processing involves interaction in two senses: an interaction between cognition, language and sensorimotor systems within the individual as well as between individuals involved in interpersonal communication. A deficient or reduced functioning in any of these systems may result in limitations in communicative choices and use of compensatory strategies (Perkins 2007). This complex interaction of systems generates unique effects for each individual and for each communicative event or genre. Therefore, we cannot predict what problems or skills will persist or even emerge, only that profiles change over time.

The type of data used to generate SELIMI in this study, a picture elicited narrative task, is a fairly straightforward task to administer, and well-known by speech-language pathologists. It also represents an ecologically valid task that has been used in many studies of different populations and languages (e.g., Berman and Slobin 1994). It is a task that children in early school age in general perform well and is predictive of academic success. However, the time required for transcription and subsequent analyses may present a challenge in the clinical context.

LSA has so far not been applied to clinical language samples, but mostly to carry out automatic scoring of written texts. High agreement has been found between automatic scoring and human rating of text quality (e.g., Landauer et al. 1997; Foltz et al. 1999). These results are much in agreement with the results of the present study, as well as with Hansson et al. (2016). We agree with McNamara (2011), who concludes that models like LSA are able to simulate human knowledge and they can be used to extract semantic representations, but that they need to be complemented with other approaches.

Another point we want to emphasize is that diagnostic accuracy may not best be reached by a measure of linguistic (e.g., semantic) maturity. For example, evaluations of the validity of different lexical-semantic measures (mainly lexical diversity) for differentiating DLD from controls on a group level varies. Several studies find that the measure D (e.g., Klee et al. 2004; Owen and Leonard 2002; Ooi and Wong 2012) is fairly accurate in identifying children with DLD. As mentioned in the introduction, there is an impressive amount of studies related to the validity of different lexical-semantic measures and to what extent they are confounded by other factors. One possible influencing factor that is often discussed is text length. For example, type-token ratio is a measure clearly influenced by length as measured in total number of words (Richards and Malvern 1997). Children with language difficulties often produce short samples, therefore, it is reassuring that short samples seem to be sufficient (see e.g. Heilmann et al. 2010). This is also the case for SELIMI. Many of the narratives in the present study were short, about half of the narratives were shorter than 100 words. This is important when studying children with language difficulties at text level, when texts of more than 100 words may be hard to elicit.

We emphasize the need of further studies replicating the results on larger samples with the aim to refine the method. Also, further studies based on other types of data (e.g., word fluency or word association tasks; Roll et al. 2011) and with more specific semantic questions are needed. Furthermore, individual variation is large within the group of children with DLD. For this study there were no criteria as to difficulties within specific aspects of language. For example, important insight would be gained if we include children with documented specific difficulties in the lexical-semantic domain.

Our purpose here was to examine possible diagnostic accuracy of the measure SELIMI. In the future it should be related to other variables from analysis of narratives, not only of linguistic (grammatical, semantic-lexical) quality but also to text quantity. Although a range of steps have to be taken before LSA can be used clinically, we believe that it has a potential for assessment of children with, or with suspected, language disorder. Methodological advances in research on semantic maturity and vulnerability in school-age children are surprisingly few and there is a need for frameworks representing’ out of the box’ thinking, as in the work reported here.

### Methodological Considerations: Influencing Factors

When interpreting the results, it is important to take into consideration that the data sets are limited to one type of narrative. In the present study we used a spoken picture elicited narrative task, which is a fairly straightforward task for typically developing children this age. It would be interesting to verify the results using other types of narrative tasks, such as personal narratives, or using another genre, such as expository language, which is more demanding when assessing school-children older than eight. A comparison between spoken and written narratives would also be interesting to increase our comprehension of the discriminative ability of SELIMI and, in the long run, its relevance for assessment. A related factor is the sampling context, for example a conversational context versus narratives. Heilmann et al. (2010) found no effect of sampling context in comparing two age-groups of children with typical language development. Similarly, Kover et al. (2012) found no significant effect of context on lexical diversity also measured as number of different words in data from adolescents with fragile X syndrome, Downs syndrome and typically developing 3–6-year-olds.

Another potential limitation is that the corpus used for generating the semantic representation is from a different type of text (newspaper texts), and thus based on a different vocabulary than the narratives analysed here. However, earlier studies indicate that the quality of the semantic representation is more dependent on the size of the corpus than on close matching of the topic (Garcia and Sikström 2014).

A common conclusion in studies of lexical-semantic properties at the text level is that there is a need for more studies investigating what factors influence the different measures and that evaluations provide important information for clinicians and researchers to take into account when selecting measures of lexical diversity in clinical data (Fergadiotis et al. 2015).

## Conclusion

In the present study we have followed up on the previous findings in Hansson et al. (2016) by creating a new LSA based method, SELIMI, to discriminate between children with DLD and typically developing controls. In the present study as well as in the previous study the LSA based scores show good correspondence with outcome, in the first study regarding developmental maturity, and in the second regarding clinical diagnosis. The results indicate that semantic difficulties in children with DLD can be shown to be manifested at text level by SELIMI. SELIMI has today no direct clinical applicability but has the potential to contribute to semantic assessments to incorporate the quality of semantic networks in addition to traditional measures focusing on the word level (McGregor 2009; Sheng and MacGregor 2010). Better understanding of semantic development and theoretically based measures of semantic development to inform assessment and intervention are strongly called for. Hopefully our study can inspire further studies on this topic.
